# Speech disturbance at stroke onset is correlated with stroke early mortality

**DOI:** 10.1186/1471-2377-13-87

**Published:** 2013-07-15

**Authors:** Kazuo Shigematsu, Hiromi Nakano, Yoshiyuki Watanabe, Tatsuyuki Sekimoto, Kouichiro Shimizu, Akihiko Nishizawa, Atsushi Okumura, Masahiro Makino

**Affiliations:** 1 Department of Neurology, National Hospital Organization, Minami Kyoto Hospital, Kyoto, Japan; 2 Department of Neurosurgery, Kyoto Kidugawa Hospital, Kyoto, Japan; 3 Department of Epidemiology for Community Health and Medicine, Kyoto Prefectural University of Medicine, Graduate School of Medical Science, Kyoto, Japan; 4 Department of Neurosurgery, Kyoto Prefectural Yosanoumi Hospital, Kyoto, Japan; 5 Department of Neurosurgery, Kyoto Fushimi Shimizu Hospital, Kyoto, Japan; 6 Department of Internal Medicine, The Nishizawa Clinic, Kyoto, Japan; 7 Department of Neurosurgery, Jujyo Rehabilitation Hospital, Kyoto, Japan; 8 Department of Neurology, Japanese Red Cross Kyoto Daini Hospital, Kyoto, Japan

## Abstract

**Background:**

Speech disturbance is a common symptom of stroke and is important as a prompt identifier of the event. The frequency of the symptom among each stroke subtype, differences between patients with and without speech disturbance and its correlation to early mortality remain unclear.

**Methods:**

The Kyoto prefecture of Japan has established a registry to enroll new stroke patients in cooperation with the Kyoto Medical Association and its affiliated hospitals. It is named the Kyoto Stroke Registry (KSR). We confirmed the existence or absence of speech disturbance in 1693 stroke patients registered to the KSR and investigated associations between speech disturbance and other characteristics.

**Results:**

Speech disturbance was observed in 52.6% of cerebral infarction (CI), 47.5% of cerebral hemorrhage (CH), and 8.0% of subarachnoid hemorrhage (SAH) cases. Characteristics showing statistically significant differences between patients with and without speech disturbance and patients were age, blood pressure, history of hypertension, arrhythmia and diabetes mellitus, habit of tobacco and alcohol, and paresis. Mortality rates of patients with/without speech disturbance were 5.2%/1.2% for CI, 12.5% /4.1% for CH, and 62.5%/ 9.0% for SAH. Adjusted hazard ratios were 2.63 (1.14-6.13, p = 0.024) in CI, 4.15 (1.41-12.23, p = 0.010) in CH, and 20.46 (4.40-95.07, p < 0.001) in SAH).

**Conclusion:**

Speech disturbance was frequently observed in stroke patients at the onset and therefore could be useful to identify the problem at the earliest stage. Hazard ratio for death was higher in stroke patients with speech disturbance than patients without. Speech disturbance is a prompt predictor of stroke early mortality.

Hiromi Nakano, Yoshiyuki Watanabe, Tatsuyuki Sekimoto, Kouichiro Shimizu, Akihiko Nishizawa, Atsushi Okumura and Masahiro Makino contributed equally to this work.

## Background

Speech disturbance is one of the commonest symptoms in stroke [1,2]. It is important as a prompt identifier of the event [3]. Speech disturbance is often noticeable for patients themselves, as well as people with them at the onset of stroke. This may urge them to call ambulances and help them to medical care sooner. It is used among ambulance paramedics in order to identify an acute onset of stroke even before hospital arrival. For example, the FAST assessment, consisting of facial weakness, arm weakness and speech disturbance, was developed as a stroke identification instrument [4,5].

Speech disturbance should be also important as a prompt assessment of stroke severity if its correlation to early mortality is proved. The relationship between speech disturbance and stroke outcome remains unclear. Hazard ratio for death comparing patients with speech disturbance and without speech disturbance is not known.

The relationship between the symptoms at the onset of a stroke and early mortality should provide valuable information to identify patients who could benefit most from intensive care at the earliest stage of the disease [6-8]. Goldstein et al. reported that speech disturbance could be used to measure the severity of stroke [9]. Rothwell et al. used speech disturbance to identify a high early risk of stroke after transient ischemic attack [10]. Still, the frequency of speech disturbance at the onset of stroke is controversial. Also, statistical data on the characteristics of onset stroke patients with speech disturbances are rare.

We studied speech disturbances by reviewing a very large stroke registry, which should provide to grasp the outlines of this still unsolved issue.

The hypothesis of this study is that speech disturbance noticed by patients and their bystanders, and confirmed by paramedics and physician, may correlate with stroke severity and could therefore be a prompt predictor of outcome [11].

The aims of the study are to clarify the following three points: firstly, how frequently speech disturbance occurs in stroke, secondarily, how it is related to the patients’ other present and past health conditions and, lastly, whether it correlates to early mortality rate.

## Methods

The Kyoto prefecture of Japan has established a registry to enroll all new stroke patients in cooperation with the Kyoto Medical Association and its affiliated hospitals with help from the data collecting agency known as the Kyoto Stroke Registry (KSR). In this study, we reviewed the KSR and confirmed the existence or absence of speech disturbance in stroke patients identified from January 1999 to December 2000 in the KSR [12]. The diagnosis of stroke was done by local neurologists and/or neurosurgeons based on the WHO definition [13]. Inclusion and excluding criteria for the KSR were described elsewhere [14,15]. Each registry recorded age, sex, date of stroke onset, blood pressure and arrhythmia on arrival, history of hypertension, arrhythmia, diabetes mellitus and hyperlipemia, use of tobacco and/or alcohol, type of paresis, consciousness level, delay time, and clinical outcome 30 days after the onset.

In cases where patients died within a month, the durations of the patients’ survival were also recorded. We classified the patients into CI, CH, SAH and others by neurological examination and the findings of CT scans (n = 1,515, 89.5%), MRI scans (n = 1,080, 63.8%), and angiographies (n = 432, 25.5%).

The presence of a speech disturbance at the onset of the stroke was determined by the local neurologists and/or neurosurgeons by an assessment of speech fluency and clarity during conversation with the patients at the first medical examination in the emergency room [16,17] and by the information from the patients and people who were with the patients at the time. We regarded slurred and non fluent speech as speech disturbance. Patients who were difficult to assess for some reason including a depressed consciousness level were recorded as ‘unconfirmed” and were excluded from the further analyses. For the purpose of pragmatic use at emergency setting, speech disturbance was considered as a uniform feature, without making a distinction among detailed forms such as dysarthria and dysphasia. Systolic and diastolic hypertension was defined when blood pressure was 140 mmHg and 90 mmHg or higher. Arrhythmia was diagnosed as such by physical examination and electrocardiogram. Hyperlipemia was defined when serum cholesterol level was 220 mg/dl or higher and/or triglyceride 150 mg/dl or higher.

History of hypertension, arrhythmia, hyperlipemia and diabetes mellitus was based on information from the patients themselves or their families, their medical records when available and medicines that the patients had been taking. Former smokers, who had quit smoking more than a year, were classified as patients without tobacco use.

### Ethics statement

This research was performed in accordance with the ethical principles for medical research involving human subjects outlined in the Declaration of Helsinki. This was approved by the Board of Directors, the Kyoto Medical Association, the Department of Health and Welfare, Kyoto Prefecture and Ethics Committee of the National Hospital Organization, Minami Kyoto Hospital. Since all identifying personal information was stripped from the secondary files before analysis, the boards waived the requirement for written informed consent from the patients involved.

### Statistical analysis

The frequencies of speech disturbance among the three stroke types were determined and evaluated for univariate associations by the Chi-square analysis with the Bonferroni correction. Numerical data such as age and blood pressure were compared with the Student-t test with the Bonferroni correction. Each characteristic of patients with speech disturbance and that of patients without such speech disturbance were compared by the Fisher’s exact test for categorical data and by the Student-t test for numerical data. We examined the associations of each characteristic with speech disturbance by a bivariate logistic regression analysis. A logistic regression model was used to estimate odds ratios for death, comparing those with and without characteristics. We used the Cox proportional hazard regression to calculate unadjusted and adjusted hazard ratios and their 95% confidence intervals for the risk of death. We generated Kaplan-Meier curves of estimated hazard function. Comparisons between patients with speech disturbance and patients without speech disturbance were performed using a log-rank test. Analyses were performed using SPSS ver.19. All reported p values were 2-sided. Statistical significance was set at p < 0.05.

## Results

The presence or absence of speech disturbance was judged as such in 1,693 stroke patients. In this study cohort, 1,247 (73.7%) were CI, 339 (20.0%) were CH, and 100 (5.9%) were SAH. A small number of patients (n = 10; 0.6%) had a combination of stroke types. Characteristics of the patients are summarized in Table 1. Speech disturbance was seen in 48.9% of all stroke patients: in 52.6% of CI patients, in 47.5% of CH patients, and in 8.0% of SAH patients.

**Table 1:** **Characteristics of stroke patients** (**n** = **1**,**693**)

	**Overall**	**Cerebral infarction n = 1,247**	**Cerebral hemorrhage n = 339**	**Subarachnoid hemorrhage n = 100**
	**n = 1,693**			
**Age (SD)**	70.9 (12.2)	72.1 (11.3) ^*1*2*3^	69.4 (13.0) ^*1*2*3^	61.7 (14.2) ^*1*2*3^
**Sex, % female (female: male)**	44.8 (759:934)	41.5^*1*2*3^ (517:730)	48.1^*1*2*3^ (163:176)	62.0^*1*2*3^ (62:38)
**Systolic blood pressure (SD) mmHg**	161.7 (30.1)	159.9 (29.6) ^*1^	169.8 (31.3)^*1*2^	157.5 (28.8) ^*2^
**Diastolic blood pressure (SD) mmHg**	87.4 (17,1)	86.5 (16.5) ^*1^	91.1 (18.7) ^*1*2^	86.6 (18.9) ^*2^
**Hypertension history, % (n = with: without)**	60.6 (955: 622)	59.3^*1*2*3^ (696: 477)	70.1^*1*2*3^ (216: 92)	44.0^*1*2*3^ (40: 51)
**Arrhythmia, % (n = with: without)**	15.5 (255: 1389)	18.7^*1*2*3^ (227: 987)	7.6^*1*2*3^ (25: 306)	3.3^*1*2*3^ (3:89)
**Arrhythmia history, % (n = with: without)**	19.3 (305:1273)	23.8^*1*2*3^ (279:895^)^	7.5^*1*2*3^ (23:285)	3.4^*1*2*3^ (3:86)
**Diabetes mellitus history, % (n = with: without)**	22.5 (359:1235)	25.7^*1*2*3^ (307:886)	15.1^*1*2*3^ (46:259)	5.6^*1*2*3^ (5:84)
**Hyperlipemia history, % (n = with: without)**	19.1 (297: 1,259)	22.4^*1*2*3^ (260: 903)	10.1^*1*2*3^ (30:267)	6.7^*1*2*3^ (6:83)
**Tobacco use,% (n = with: without)**	30.7 (438:989)	32.5^*1^ (350:727)	21.3^*1*2^ (56:207)	34.9^*2^ (29:54)
**Alcohol use, % (n = with: without)**	40.3 (564:834)	39.6^*3^ (419:640)	41.1^*2^ (104:149)	48.1^*2*3^ (39:42)
**Paresis, % (n = with: without)**	75.4 (1,266:414)	79.7^*3^ (987:252)	78.6^*2^ (264:72)	11.2^*2*3^ (11:87)
**Speech disturbance, % (n = with: without)**	48.9 (828:865)	52.6^*1*2*3^ (656:591)	47.5^*1*2*3^ (161:178)	8.0^*1*2*3^ (8:92)

Data on some characteristics were missing in a few patients.

Differences statistically significant between Cerebral Infarction and Cerebral Hemorrhage, Cerebral Hemorrhage and Subarachnoid Hemorrhage, and Subarachnoid Hemorrhage and Cerebral Infarction were marked by ^*1^, ^*2^, and ^*3^ respectively*; P < 0.05. Abbreviations: *SD* standard deviation.

The characteristics of the patients, comparing patients with speech disturbance and patients without speech disturbance, are summarized in Table 2. Data on speech disturbance, age, and sex were complete in all patients. The other characteristics had missing data in a few patients (numbers are shown in Tables).

**Table 2:** Characteristics of patients with and without speech disturbance

	**With speech disturbance, n = 828**	**Without speech disturbance, n = 865**	**p**
**Age (SD)**	72.9 (11.6)	69.0 (12.3)	<0.001*
**Sex, % female (n = female: male)**	44.6 (369:459)	45.1 (390:475)	0.845
**Systolic blood pressure (SD) mmHg**	165.9 (31.1)	157.7 (28.6)	<0.001*
**Diastolic blood pressure (SD) mmHg**	88.9 (17.9)	86.0 (16.4)	<0.001*
**Hypertension history, % (n = with: without)**	63.9 (487:275)	57.4 (468:347)	0.009*
**Arrhythmia, % (n = with: without)**	19.8 (159:646)	11.4 (96:743)	<0.001*
**Arrhythmia history, % (n = with: without)**	24.3 (187:584)	14.6 (118:689)	<0.001*
**Diabetes mellitus history, % (n = with: without)**	25.9 (202:615)	19.3 (157:656)	<0.002*
**Hyperlipemia history, % (n = with: without)**	18.8 (142:615)	19.4 (155:644)	0.796
**Tobacco use, % (n = with: without)**	27.7 (196:511)	33.6 (242:478)	0.016*
**Alcohol use, % (n = with: without)**	35.1 (242:448)	45.5 (322:386)	<0.001*
**Paresis, % (n = with: without)**	89.7 (735:84)	61.7 (531:330)	<0.001*

Data on some characteristics were missing in a few patients. *P < 0.05.

The patients with speech disturbance were older, higher in a female/male ratio, higher in systolic and diastolic blood pressure, and more often associated with history of arrhythmia and diabetes mellitus.

The odds ratios for speech disturbance, comparing those with and without various characteristics, and the p values estimated by a logistic regression analysis are summarized in Table 3.

**Table 3:** **Odds ratios for speech disturbance**, **comparing those with and without characteristics listed in the left column of the table**

	**Odds ratio**	**95% Confidence interval**		**p**
		**Lower**	**Upper**	
**Age (70 or over)**	0.88	0.68	1.13	0.302
**Sex (Female)**	0.64	0.49	0.84	0.001
**Systolic hypertension (140 or over), mmHg**	0.81	0.60	1.11	0.187
**Diastolic hypertension (90 or over), mmHg**	1.19	0.91	1.56	0.196
**Arrhythmia**	1.32	0.75	2.32	0.335
**Hypertension history**	0.98	0.75	1.28	0.897
**Arrhythmia history**	1.20	0.71	2.01	0.495
**Diabetes mellitus history**	1.21	0.90	1.63	0.202
**Hyperlipemia history**	0.80	0.59	1.10	0.168
**Tobacco use**	0.76	0.56	1.02	0.064
**Alcohol use**	0.50	0.37	0.66	<0.001
**Paresis**	2.83	2.15	3.73	<0.001
**Cerebral infarction**			reference	<0.001*
**Cerebral hemorrhage**	0.75	0.55	1.04	0.083
**Subarachnoid hemorrhage**	0.18	0.07	0.42	<0.001

*: stroke subtypes as a whole.

**Figure 1:**
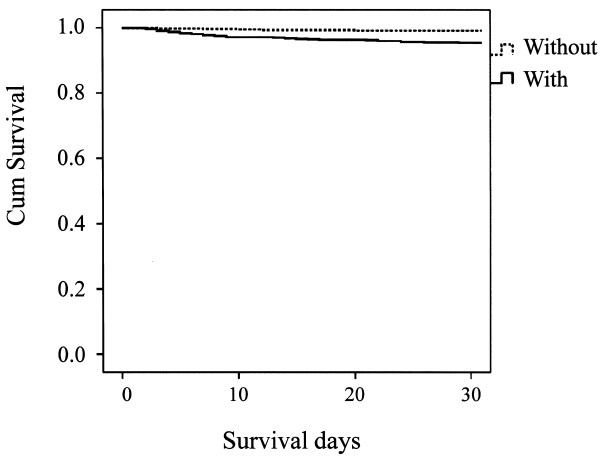
Kaplan-Meier estimated survival curves, comparing patients with speech disturbance and without speech disturbance in cerebral infarction.

**Figure 2:**
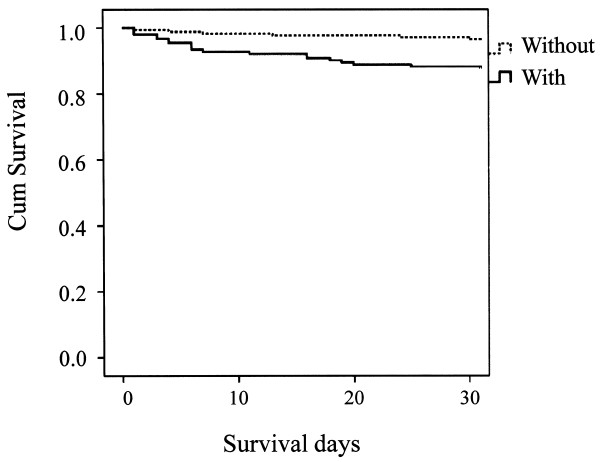
Kaplan-Meier estimated survival curves, comparing patients with speech disturbance and without speech disturbance in cerebral hemorrhage.

**Figure 3:**
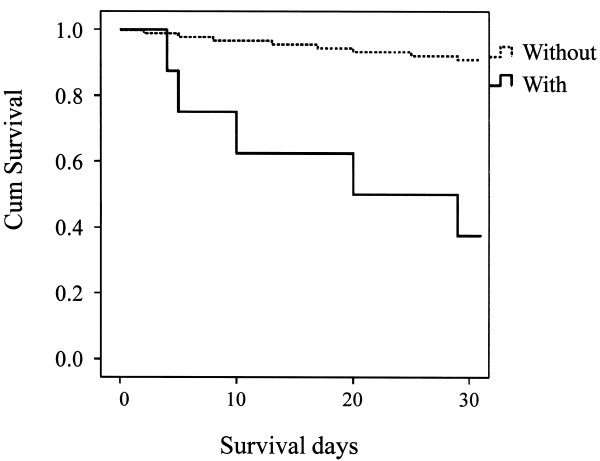
Kaplan-Meier estimated survival curves, comparing patients with speech disturbance and without speech disturbance in subarachnoid hemorrhage.

A total of 79 out of 1,623 (4.9%) stroke patients died within 30 days after the onset of stroke (data on 70 patients (4.1%) were missing). Forty out of 1,159 (3.3%) died in CI cases, 26 out of 321 (8.1%) died in CH cases, and 13 out of 97 (13.4%) died in SAH cases. Kaplan-Meier estimate survival curves, comparing patients with speech disturbance and without speech disturbance are presented in Figures 1, 2 and 3. A log-rank test supported the statistical significance of differences in survival between patients with and without speech disturbance in all three stroke categories (p < 0.001 in stroke as a whole, p < 0.001 in CI, 0.006 in CH, and <0.001 in SAH).

Hazard ratios for death, comparing patients with speech disturbance and those without, with 95% confidential intervals are shown in Table 4. Speech disturbance at stroke onset showed an association with death within 30 days after the onset of stroke.

**Table 4:** **Hazard ratios for death**, **comparing patients with speech disturbance and those without speech disturbance**

	**Hazard ratio**	**95% Confidence ratio**		**P**
		**Lower**	**Upper**	
Overall (OA) *1	2.79	1.71	4.56	<0.001
Cerebral infarction (CI) *1	4.36	1.93	9.86	<0.001
Cerebral hemorrhage (CH) *1	3.17	1.33	7.54	0.009
Subarachnoid hemorrhage (SAH) *1	9.62	3.13	29.57	<0.001
OA*2	2.81	1.71	4.62	<0.001
CI*2	3.93	1.73	8.94	0.001
CH*2	3.17	1.33	7.57	0.009
SAH*2	10.59	3.25	34.54	<0.001
OA*3	2.55	1.47	4.44	0.001
CI*3	2.64	1.14	6.13	0.024
CH*3	4.15	1.41	12.23	0.010
SAH*3	20.46	4.40	95.07	<0.001

*1: Unadjusted.

*2: Adjusted for age and sex.

*3: Adjusted for age, sex, systolic and diastolic blood pressures, arrhythmia and paresis.

## Discussion

This study showed two major things. Firstly, speech disturbance was frequently observed in stroke patients at the onset and therefore could be useful to identify the problem at the earliest stage. Secondarily, patients with speech disturbance had a higher mortality at 30 days after the stroke and speech disturbance could therefore be useful as one of the earliest tools to assess the severity of stroke. Paying more attention to speech disturbance should contribute to giving patients earlier access to hospitals and the appropriate immediate care of medical staff [18].

Speech disturbance was seen in about half of all stroke patients, which agrees with previous reports that it is one of the commonest symptoms of stroke. The study added information on the frequencies of the symptom in CI, CH, and SAH, respectively, and clarified differences in characteristics between patients with and without speech disturbances. Reports on the frequencies of speech disturbance in stroke vary widely. Jerntorp et al. reported that they observed a speech deficit in 26.1% of ischemic stroke patients and in 12.5% of hemorrhagic stroke patients [2]. Kothari et al. reported that they observed a speech abnormality in 11% at the onset of stroke [19]. Macdonell et al. reported dysarthria in 60% of cerebellar infarction patients [20].

The reasons why it is difficult to define the frequency of speech disturbance in stroke and to investigate its relation with mortality presumably include the followings. First, speech disturbance is a rather broad term, probably ranging from dysarthria to dysphasia, and may be difficult to assess in a detailed manner especially at emergency settings. Second, speech disturbance is a symptom that possibly fluctuates during emergency care.

In this study, patients with slurred and non fluent speech at the emergency room were regarded as patients with a speech disturbance. Since the speech disturbance researched here depended on information from patients and paramedics, it should be rather suitable to confirm its practical importance as a prompt identifier of stroke and as a prompt predictor of stroke outcome.

The present study showed that speech disturbance is more common in CI and in CH than in SAH. One possible explanation of this difference is that SAH is caused by the rupture of an aneurysm of the cerebral artery in the subarachnoid space outside of the brain cortex, and therefore should affect the brain in a rather indirect manner.

Odds ratios for speech disturbance, comparing those with and without various characteristics, past histories and paresis, are summarized in Table 3.

The most remarkable finding is the constant association of speech disturbance with early mortality in stroke as a whole, as well as in all three major subtypes of stroke. Although physicians empirically know that speech disturbance correlates with a poor outcome of stroke, there has been no quantitative data on the correlation with mortality.

Hazard ratios for death within 30 days after stroke onset in patients with a speech disturbance were much higher than those in patients without such speech disturbance. The statistical significance remained after adjustment for age, sex, systolic and diastolic blood pressures, arrhythmia and paresis. Therefore, speech disturbance at the onset of stroke can predict early stroke mortality, independent of age, sex, blood pressures, arrhythmia and paresis.

Outcomes and their predicting factors are great concern in stroke care. Various factors have been investigated and clarified the correlation to the outcome. The present study confirmed the validity of speech disturbance as a predicting factor, especially at emergency care.

### Limitations

First, this study did not cover treatments such as thrombolysis for CI, which should affect the outcome. Treatments vary widely depending on various factors. It is virtually impossible to adjust them in the population-based study. However, thrombolysis has no indication for hemorrhagic stroke and surgery has rarely done for ischemic stroke. This study showed patients with speech disturbance had higher hazard ratios for death in both CI and CH.

Second, detailed language examinations are difficult at emergency care, and therefore speech disturbance was not linguistically classified in the study. Speech disturbance noticeable by ordinary people and confirmable by paramedics and physicians should have practical importance. The present study clarified the usefulness of such speech disturbance as a prompt indicator of stroke severity in a pragmatic manner.

Third, some patients were unable to be assessed as to the presence or absence of speech disturbance, for some reason including conscious disturbance. Consciousness disturbance, however, is well established as a predictor of a poor outcome of stroke. Prompt predictors of the mortality in stroke patients without consciousness disturbance are of importance.

Fourth, we analyzed all death up to 30 days after the stroke onset rather than death long after the event because we thought early mortality should reflect well deaths rather directly associated with stroke.

With all these limitations, however, the study is based on a large number of patients and a significant bias affecting the major conclusions is unlikely.

## Conclusions

Speech disturbance was frequently observed in stroke patients at the onset and therefore could be useful to identify the problem at the earliest stage. It could also be a prompt predictor of the outcome.

## Competing interests

All authors have no competing interests.

## Authors’ contributions

All authors contributed equally in the data collection. KS conceived the idea of the study and was responsible for the data analysis and produced the tables and graphs. HN was a co-investigator and Chair of the Kyoto Stroke Registry Committee (KSRC). YW was a co-investigator and vice Chair of the KSRC. KS, TS, KoS, AN, AO and MM were members of the KSRC. All authors read and approved the final manuscript.

## Pre-publication history

The pre-publication history for this paper can be accessed here:

http://www.biomedcentral.com/1471-2377/13/87/prepub
